# Brillouin microscopy analysis of the fibroblast mechanical response to substrate's stiffness

**DOI:** 10.1039/d5sm00315f

**Published:** 2025-05-13

**Authors:** Vsevolod Cheburkanov, Vladislav V. Yakovlev

**Affiliations:** a Department of Biomedical Engineering, Texas A&M University College Station TX USA yakovlev@tamu.edu; b Department of Physics and Astronomy, Texas A&M University College Station TX USA

## Abstract

Cancer mechano-adaptation remains poorly understood due to the lack of imaging technologies capable of quantifying both mechanical and biochemical properties of cells and their microenvironment in 3D culture and *in vivo*. This challenge arises primarily due to the invasiveness of existing mechanical measurement techniques and their inability to assess mechanical properties in highly heterogeneous structures such as living tissues. Brillouin microscopy is an emerging, label-free technique that enables measurements of local mechanical properties with microscopic spatial resolution. In this study, we non-invasively imaged the elastic properties of monolayer 4T1 murine fibroblast cells using Brillouin microscopy and analyzed their response to variations in the mechanical properties of the external environment. Our findings demonstrate a significant correlation between the mechanical properties of the extracellular matrix and cancer cells, as assessed through Brillouin microspectroscopy in a non-invasive and safe manner. These results highlight the potential of Brillouin spectroscopy as a robust and effective technique for the characterization of biomechanical properties in cancer cells, offering valuable insights into their mechanical behavior.

## Abstract 2

Brillouin microspectroscopy is a powerful tool for probing the viscoelastic properties of cells, revealing a strong and direct correlation between cellular mechanics and the mechanical characteristics of their extracellular environment.

## Graphical abstract

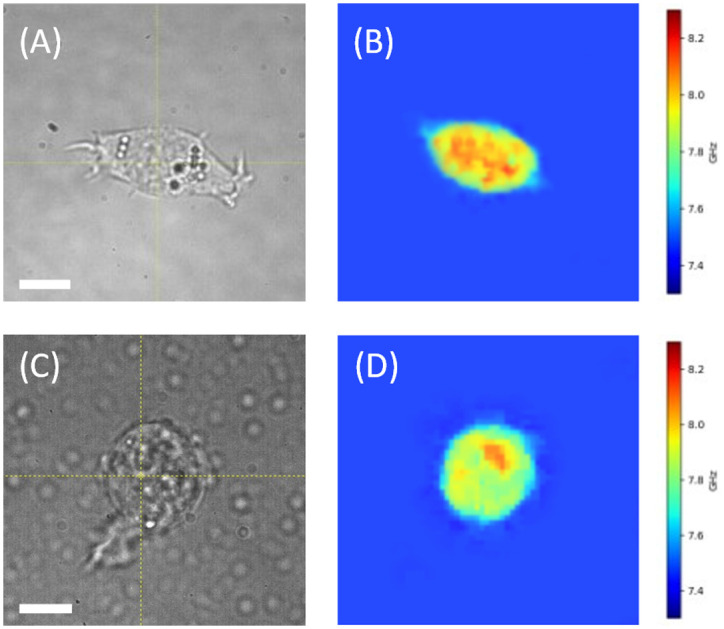

## 1. Introduction

The resistance of cancer cells to chemotherapy and radiation therapy is caused in part by the local adaptation of cancer cells to the tissue microenvironment *in vivo*.^1–3^ Because this adaptation occurs in a spatial and temporal context, a complete understanding of the process necessarily requires the use of 3D culture systems and intravital imaging approaches for organotypic tracking of cancer cell response and fate. A hallmark of cancer is alterations to the mechanical properties of the tumor and its microenvironment.^4,5^ The adaptation of cancer cells to mechanical challenges, which we term mechano-adaptation in this application, is important for cancer cell survival^6,7^ and resistance signaling.^8–10^ The process of mechano- adaptation is poorly studied owing in large part to a lack of imaging technologies that can quantify mechanical cell properties *in vivo*. This report is focused to address this need by optimizing Brillouin microscopy to achieve the first quantification of mechanical properties of cells, tissues and the extracellular matrix in 3D cancer models. To be useful, the mechanical measurement technique for preclinical research must be non-invasive and enable analysis of living cells in 3D tissue contexts *in vivo*. It should be quantitative. It should quantify mechanical properties not only of cancer cells and sub-cellular structures like nuclei but also cells and structures of the tumor micro-environment and of different cell types in the tumor across widely varying length scales in one measurement. Further, each imaging module should be inter-operable in that it should be easily combined with other imaging techniques such as metabolic imaging or fluorescence imaging. These criteria are not all met by currently available mechanical measurement techniques. Preclinical models are not amenable to techniques like atomic force microscopy (AFM), particle tracking microrheology (PTM), or traction force microscopy (TFM). Yet, without satisfying such criteria in preclinical animal models, the conditions supporting cancer progression and cellular responses to drugs as well as confounding events of the drug response, such as perturbation of stromal cells and vasculature, will remain difficult to determine. Brillouin microscopy (BM), a technique rooted in a century-old spectroscopic method that relies on inelastic light scattering by acoustic phonons within a medium, is experiencing its renaissance.^11^ Offering spatial resolution constrained solely by the optical diffraction limit, BM addresses a critical gap by delivering detailed information about local viscoelastic properties at a microscopic scale in a non-invasive manner.^12–16^ BM has established itself as a technique to measure high-frequency longitudinal viscoelastic moduli.^11,13–15^ Brillouin scattering is the inelastic scattering of light by sound waves through thermal or electrostrictive excitation.^17^ The incident monochromatic radiation experiences a frequency shift, *Ω* = ±2(*v*/*λ*)sin(*θ*/2), which is determined by a scattering angle, (*θ*), the speed of sound (*v*) in the sample, and the wavelength, (*λ*), of the incident radiation in the medium. For the most commonly used backscattered geometry, *θ* = 180° and, hence, *Ω* = ±2(*v*/*λ*), or *v* = *λΩ*/2. This frequency shift, often called Brillouin shift or Brillouin frequency, has been considered as a contrast mechanism for assessing local elastic properties. The linewidth of the peak, which is inversely proportional to the lifetime of the acoustic phonon, has been interpreted in terms of local viscosity.^18–22^ The complex Brillouin modulus, M* = *M*′ + *jM*′′, is calculated using a well-established relationship.^17,23–25^ The real part (or elastic component) is *M*′ = *ρv*^2^ = *ρ*(*λΩ*/2)^2^, where *ρ* is the density of material. The imaginary part (or viscous component), *M*′′ = *M*′(*Δ*/*Ω*) is proportional to the linewidth (FWHM) of the Brillouin line, *Δ*, (see^17^ for more details). It is important to note that the Brillouin shift and linewidth are related to a high-frequency viscoelastic modulus, which is different from the static one. The mechanical modulus of many soft materials follows a power-law dependence on acoustic frequency (*ω*): *G*′ = *G*_0_(*ω*/*ϕ*_0_)^*γ*^, in agreement with structural damping and soft glassy rheology models. Here, *G*_0_ and *ϕ*_0_ are scaling factors with magnitudes in the order of 100 kPa and 100 MHz, respectively, and *γ* is the scaling exponent (*γ* = 0 for purely elastic and 0 < *γ* < 1 for viscoelastic materials).^26,27^ A similar power law can also hold for the Brillouin modulus: *M*′ = *M*_0_(*ω*/*ϕ*_0_)^*β*^, where *M*_0_, *ϕ*_0_ and *β* are constant for a specific sample (*e.g.* for porcine lens, *M*_0_ 50 GPa, *ϕ*_0_ ∼ 50–100 GHz and *β*/*γ* = 0.6^18^). A relationship between Brillouin moduli and static moduli can be established for a particular system of interest. In recent years, substantial advancements have been made in the application of Brillouin microscopy (BM) to biological imaging.^11,14,15^ A well-established correlation exists between the local viscoelastic properties of biological systems and the Brillouin shift and linewidth.^20,28–38^ Although initial studies suggested that the Brillouin shift could be influenced by the water content of a sample, subsequent research has solidified the consensus that Brillouin spectroscopy accurately reflects local viscoelastic characteristics. Specifically, the Brillouin shift correlates with elastic properties, while the linewidth is indicative of viscosity. The significant potential of BM for advancing mechanobiology has been underscored in recent comprehensive reviews.^14,15^ In this report, we assess the intracellular mechanical properties of the 4T1 murine fibroblast cell line adhering to hydrogels with varying stiffness. The 4T1 cell line is extensively utilized as a breast cancer model,^39^ and the influence of the mechanical environment on cancer metastasis is well-documented.^40^ We investigate the potential of Brillouin microspectroscopy to characterize the viscoelastic properties of cell cultures, aiming to elucidate the relationship between intracellular stiffness and the stiffness of extracellular matrices in a non-invasive, label-free manner.

## 2. Materials and methods

### 2.1. Experiment design

To examine cellular responses to mechanical stimuli, two distinct sample groups were prepared and cellular mechanical properties on microscopic level were interrogated using Brillouin microspectroscopic imaging. The groups comprised 4T1 murine fibroblasts cultured on hydrogels with differing shear moduli *G*: one group on hydrogels with a low value storage modulus of *G* = 1 kPa and the other on hydrogels with a high value storage modulus of *G* = 308 kPa. These samples are henceforth referred to as “soft” and “stiff” gels, or “1 kPa” and “308 kPa” gels respectively. Cells were initially seeded onto hydrogel substrates and incubated according to established protocols at 37 °C in 5% CO_2_ atmosphere. Cultures were visually inspected at regular intervals to monitor formation of a monolayer and absence of contamination. Upon reaching ∼80% confluence, samples were transferred to a Brillouin microscope for imaging. To mitigate environmental stress during imaging, samples were placed on a heated microscope stage maintained at 37 °C. Each point on the sample was exposed to the laser for no longer than 200 ms, with laser radiation being blocked immediately after imaging the select culture region. Following imaging, cells were assessed visually for morphological alterations indicative of cellular damage or death. Fluorescent dyes were deliberately omitted to preserve cells under investigation.^41^ High irradiance within the sample plane can induce detrimental effects. This phenomenon is well-documented in the context of photodynamic inactivation of pathogens.^42,43^ To isolate substrate stiffness as the primary factor influencing cellular elasticity, imaging was conducted both in the absence of neighboring cells and with cells in close proximity to one another.

### 2.2. Hydrogel preparation

The selection of materials was based on the presence of specific binding sites that facilitate cell adhesion. In this regard, collagen hydrogels were chosen due to their known ability to provide such binding sites.^44^

Gel formulations were prepared to achieve target storage modulus values of 1 kPa and 308 kPa for the cell substrates. Gel with the storage modulus values of two orders of magnitude difference were selected for two reasons:

1. ECM stiffness values across various tissues in living organisms typically range from approximately 0.2 kPa in brain tissue^45^ and <10 kPa in healthy soft tissue^46^ to around 10^6^ kPa in bone tissue.^45^ Hence the selected storage modulus values fall into the boundaries for physiological values of ECM storage modulus.

2. Selecting substrate storage moduli two orders of magnitude apart is essential for enhancing the contrast of the collected data. Specifically, cells were anticipated to exhibit significantly greater stiffness at 308 kPa compared to 1 kPa.

Additionally, intermediate formulations with target storage moduli of 22 kPa, 46 kPa, and 52 kPa were synthesized to establish a conversion curve correlating Youngs modulus with the Brillouin shift value. A standardized protocol was employed to synthesize hydrogels using bovine type I collagen.^47^

The mechanical properties of the fabricated hydrogels were assessed using an Anton Paar Physica MCR 301 rheometer. The resulting error in the storage modulus measurements did not exceed 2% of the calculated value.

### 2.3. Culture preparation

**Fig. 1:**
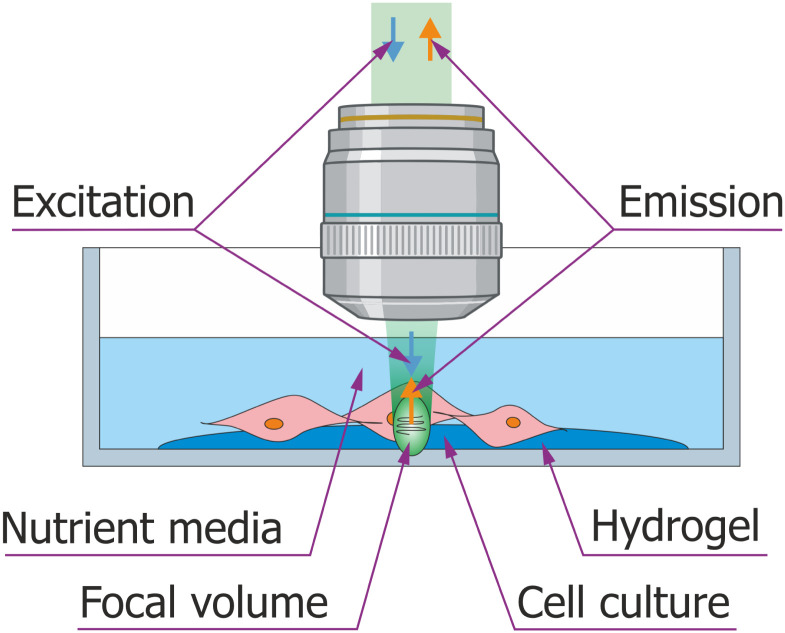
A schematic of the sample configuration used in the study. Here 4T1 murine fibroblast cells are adhered to a hydrogel substrate affixed to the bottom of the dish. Cells were cultured to ∼80% confluency, ensuring a monolayer structure, and maintained in nutrient-rich media to support metabolism and minimize stress. During imaging, the microscope objective lens was immersed into the sample from above. Acoustic perturbations (black cursive line inside focal volume) are causing excitation light (blue arrow) to inelastically scatter and emit Brillouin scattered photons (orange arrow). Detection of the signal is made in confocal configuration. Created in https://BioRender.com.

To simulate a range of extracellular matrix (ECM) stiffness values, hydrogels with two distinct elastic moduli were employed as cell substrates. Specifically, hydrogels with elastic moduli of 1 kPa and 308 kPa were selected to minimize experimental errors, maximize data contrast, and replicate a broad spectrum of cell adhesion conditions encountered *in vivo*. A schematic representation of the typical sample configuration is provided in Fig. 1.

Samples were imaged when ∼80% confluence was reached and cells covered the hydrogel surface in a monolayer structure. During imaging, cells were maintained on a heated microscope stage at 37 °C. To ensure sustained culture viability during the experiment, nutrient media was replaced between each acquisition run with fresh from the vial maintained at 37 °C.

### 2.4. Setup description and acquisition settings

**Fig. 2:**
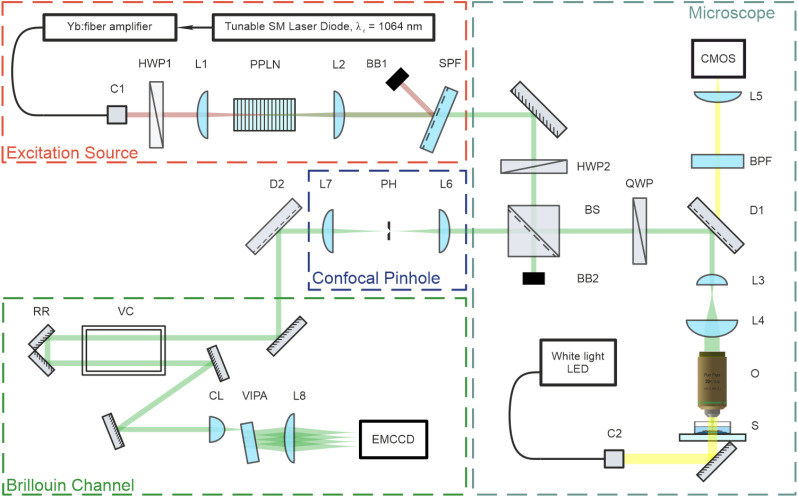
Brillouin confocal microspectrometer layout. HWP – half-wave plate, BB – beam block, BS – polarizing beamsplitter cube, QWP – quarter-wave plates, D – dichroic mirror, O – microscope objective lens, S – sample, C – fiber collimator, BPF – bandpass filter, L – plano-convex lens, PH – precision pinhole aperture, VC – iodine vapor cell, CL – cylindrical lens, VIPA – virtually imaged phase array.

Elasticity data were acquired using a custom-built upright confocal Brillouin microspectrometer configured in a backscattering geometry.^38,41,48–50^ The schematic of this setup is depicted in Fig. 2. The system comprises four primary subassemblies: an excitation source, a microscope, a confocal pinhole assembly, and the custom-built Brillouin microspectrometer.

#### 2.4.1. Excitation

A custom-built 532 nm laser with a linewidth of less than 1 MHz was utilized as the excitation source. This wavelength was produced as the second harmonic of 1064 nm laser radiation within a periodically poled LiNbO_3_:MgO crystal Covesion Ltd. The 1064 nm light was generated by a tunable single longitudinal mode laser diode (Koheras Adjustik Y10, NKT Photonics) and subsequently amplified using an Yb-doped fiber amplifier (Koheras Boostik HPA Y10, NKT Photonics).

#### 2.4.2. Microscope

The microscope body was custom-designed using standard optomechanical components (Thorlabs). The power delivered to the sample was regulated by a 532 nm half-wave plate (Thorlabs) in conjunction with a polarizing beamsplitter cube (BS, Thorlabs PBS251). A polymer quarter-wave plate was employed to achieve orthogonal polarization between the incident and scattered light, allowing the scattered signal to pass through the PBS towards the confocal pinhole assembly. Imaging was performed with a microscope objective (water immersion Nikon CFI60, 60x) featuring an effective numerical aperture (NA) of 1.0, which simultaneously delivered approximately 5 ± 0.3 mW of 532 nm radiation to the sample and collected the scattered photons. Target acquisition within the field of view was facilitated by a camera coupled to a Nikon tube lens. Transparent specimens were illuminated in transmission mode using a fiber-coupled 6000 K LED (Mightex). The sample's position within the field of view was precisely adjusted using a microscope stage with nanometric resolution capabilities (MCL Nano-LPS for fine adjustments and a custom MCL MicroStage for coarse positioning), and the system was equipped with a microscope slide warmer.

#### 2.4.3. Confocal pinhole assembly

The confocal pinhole assembly was custom-built using standard optomechanical components (Thorlabs, National Aperture) to spatially section the investigated sample. The pinhole diameter was chosen to be smaller than one Airy unit, thereby optimizing axial and lateral resolution, power throughput of the system and minimizing acquisition time of the Brillouin scattering signal.

#### 2.4.4. Custom-built Brillouin spectrometer

The output from the pinhole filter was coupled to the entrance pupil of a custom-built Brillouin spectrometer. To suppress elastically scattered photons, an iodine vapor cell (VC) (Thorlabs) was employed, heated to 70 °C. By utilizing a double-pass beam propagation geometry and fine-tuning the laser output wavelength to align with the strongest absorption band of molecular iodine, suppression ratios exceeding 40 dB were achieved. Optimal absorption occurred at a wavelength of 531.9363 nm, corresponding to line 638 with a wavenumber of 18 799.244 cm^−1^.^51^ To optimize the contrast of the recorded spectra, spectral contributions from elastically scattered photons were entirely attenuated, thereby isolating the inelastically scattered signals and enhancing the detection of relevant spectral features, minimizing the acquisition time required to detect the signal.

The Brillouin scattering signal was analyzed using a high-dispersion, custom-built single-stage VIPA spectrometer. The VIPA (OP-6721-3371-2, Lightmachinery Inc.) was specifically optimized for 532 nm and featured a free spectral range of 29.98 GHz (1 cm^−1^). Signal spectra were recorded with a water-cooled EMCCD camera (Andor Newton 970P, Oxford Instruments).

System instrumental error did not exceed 20 MHz. The signal processing algorithm utilized was detailed extensively in prior publications.^52,53^

## 3. Results and discussion

Live 4T1 murine fibroblast cell cultures, proliferating on hydrogels with elastic moduli of 1 kPa and 308 kPa, as well as cell-free hydrogels, were imaged under controlled conditions at 37 °C without CO_2_ content control. The lateral spatial sampling interval was set to 0.8 μm. To minimize the impact of thermal stress on the samples, only a single image was acquired per field of view during each imaging session.

The results of these observations are presented in the following section.

### 3.1. Cell-free hydrogel measurement with the Brillouin spectrometer

**Fig. 3:**
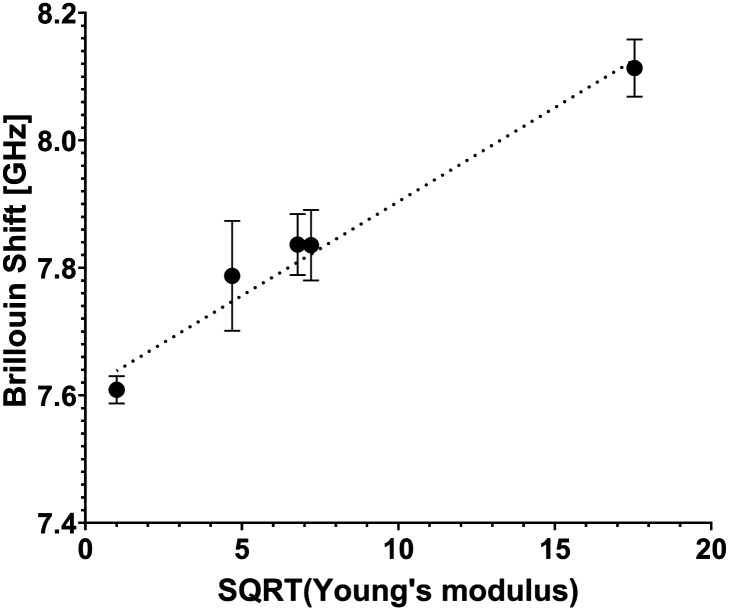
Retrieved Brillouin shift value plot *versus* square root value of storage (Young's) modulus *E*′. Cell-free hydrogels of various formulations (stiffness values of 1 kPa, 22 kPa, 46 kPa, 52 kPa and 308 kPa) were interrogated to make the scatterplot. A linear trendline is presented to show good agreement with the equation 
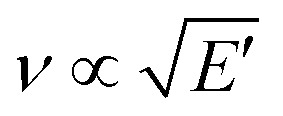
, and combined with analytical approximation 
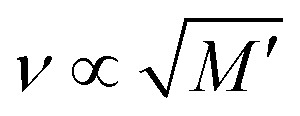
 it can be concluded that the following is correct for these hydrogels: *M*′ ∝ *E*′.

Hydrogels submerged in phosphate buffered saline (PBS) at 37 °C were analyzed using the confocal Brillouin microspectrometer. The Brillouin frequency shift values obtained in the measurements are plotted against the measured storage modulus value and are presented in Fig. 3.

According to the relation *M*′ = *ρv*^2^, where *M*′ denotes the elastic modulus, *ρ* represents density, and *ν* is the Brillouin shift value. The analytical approximation suggests that 
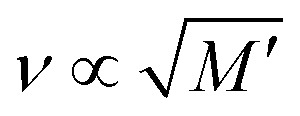
. Consequently, plotting the frequency shift against the square root of the storage modulus reveals a linear relationship, as seen in Fig. 3. This linear dependence is consistent with the analytical approximation described earlier. This finding enables the conversion of Brillouin shift values into approximate storage modulus values, thereby providing enhanced contrast for analysis.

Considering the instrumental error as a benchmark for system sensitivity, it can be inferred that the imaging system achieves a discrimination accuracy of no less than 20 kPa for transparent samples.

Error bars depicted in Fig. 3 indicate the presence of subtle microscopic inhomogeneities within the hydrogels.

The high sensitivity of the system enabled precise differentiation of various substructures within the imaged cells, while maintaining their overall integrity.

### 3.2. Cell elasticity measurement with the Brillouin spectrometer

The readability of the cell stiffness maps was enhanced through Gaussian blurring, which was applied to smooth sharp transitions between data pixels.

**Fig. 4:**
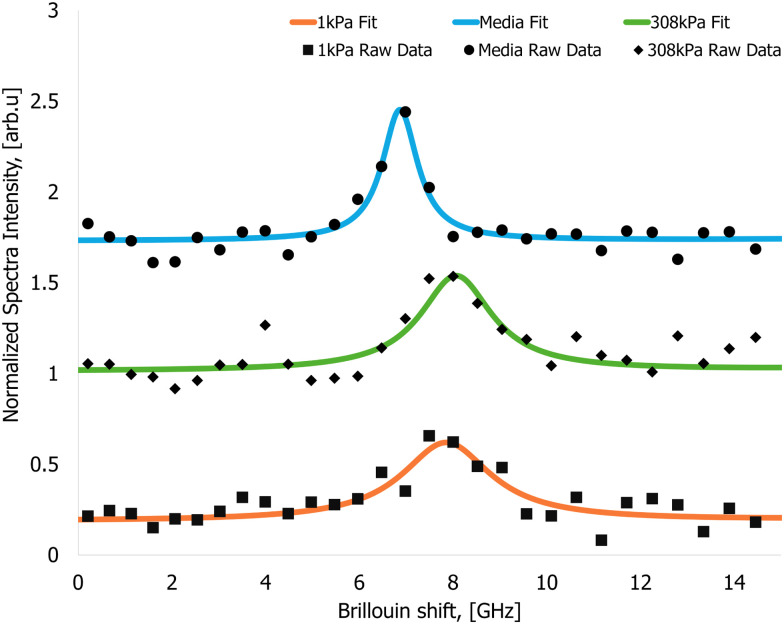
Arbitrarily picked Brillouin spectral data recorded from the samples. Brillouin spectra from the nutrient media (circles – raw signal, solid blue line – Lorentzian fit, Brillouin shift value 7.47 ± 0.07 GHz), cells cultured on a 1 kPa gel (square – raw signal, solid orange line – Lorentzian fit, Brillouin shift value 7.92 ± 0.04 GHz) and cells cultured on a 308 kPa gel (diamonds – raw signal, solid green line – Lorentzian fit, Brillouin shift value 8.05 ± 0.08 GHz).

An exemplary Brillouin-shifted spectral line obtained from the cells is presented in Fig. 4. The acquired spectral data were subjected to curve fitting, using a Lorentzian function to accurately model spectral line associated with the Brillouin scattering inside the sample.

#### 3.2.1. Single cell experiment

**Fig. 5:**
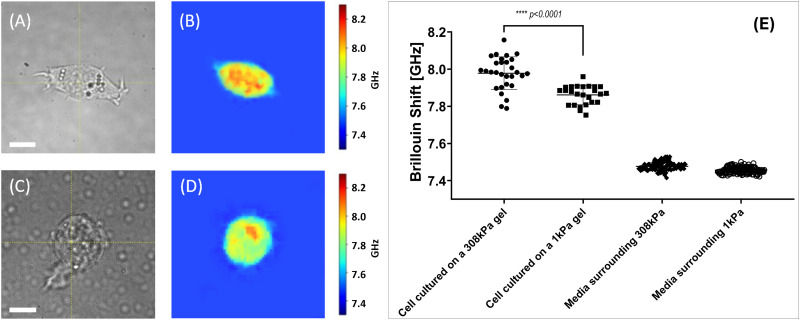
Results of the stiffness measurement experiment with the isolated cells. Panels (A) and (C) display wide-field microscope images of an isolated 4T1 fibroblast on 308 kPa and 1 kPa substrates respectively (60× magnification). White bar 10 μm. Panels (B) and (D) display Brillouin shift value maps of cultures presented in panels (A) and (B) respectively. Panel (E) displays statistics of the Brillouin shift values retrieved from the isolated cells cultured on different substrates. Here, cells grown on 308 kPa hydrogel substrates exhibited the average Brillouin shift value of 7.89 ± 0.09 GHz. Cells grown on 1 kPa hydrogel substrates exhibited the Brillouin shift value of 7.86 ± 0.05 GHz. Data sets are significantly different (*p* < 0.0001, Mann–Whitney test). Brillouin shift values retrieved from the media surrounding the 308 kPa and 1 kPa hydrogels is 7.48 ± 0.02 GHz and 7.46 ± 0.02 GHz. Presented data serves proof that cells cultured on the stiffer hydrogel substrate display a significantly larger Brillouin shift value, corresponding to higher stiffness.

Fig. 5 presents representative images of standalone 4T1 murine fibroblasts adhered to hydrogel substrates. Widefield images shown in Fig. 5A and C reveal distinct morphological differences between cells cultured under identical incubation conditions.

Fibroblasts grown on 308 kPa hydrogels exhibit an elongated morphology with prominent protrusions, whereas cells cultured on 1 kPa hydrogels display a more rounded morphology with minimal protrusions. Additionally, it was observed that fibroblasts adhere more effectively to stiffer substrates; tilting of the culture dish could dislodge cells from the soft hydrogels without affecting those on the stiffer hydrogel substrates.

The Brillouin shift value maps shown in Fig. 5B and D indicate that cells attached to stiffer substrates exhibit higher Brillouin shift values, suggesting increased stiffness compared to cells on softer substrates.

Cumulative statistics for the cells imaged under described conditions are shown in 5E. Each data point signifies an average value Brillouin shift value retrieved from the cell. It was calculated as an average shift value within the area corresponding to the cell on the shift value map. The total mean and median Brillouin shift values for these sampled regions were then computed.

Sampled cells grown on 308 kPa hydrogels exhibited an average Brillouin shift value of 7.98 ± 0.09 GHz. Median Brillouin shift value for the cells cultured on stiff hydrogels is 7.98 GHz. Data for the statistical analysis were collected from 29 cells grown on 308 kPa substrates. In contrast, 25 sampled cells grown on 1 kPa hydrogels showed an average shift value of 7.86 ± 0.05 GHz with a median value of 7.87 GHz.

These findings indicate a shift value difference exceeding 0.10 GHz, which is greater than the average Brillouin shift difference of 0.02 GHz between the media surrounding 308 kPa and 1 kPa gels (7.48 ± 0.02 GHz and 7.46 ± 0.02 GHz respectively). Difference between shift values retrieved from the nutrient media can be justified as an instrumental error or a result of natural hydrogel degradation.

Presented values suggest that the subcellular structures of 4T1 fibroblasts investigated in the cells grown on different substrates exhibit significant variations in stiffness, correlating with the stiffness of the substrate (ECM).

#### 3.2.2. Cell proximity experiment

**Fig. 6:**
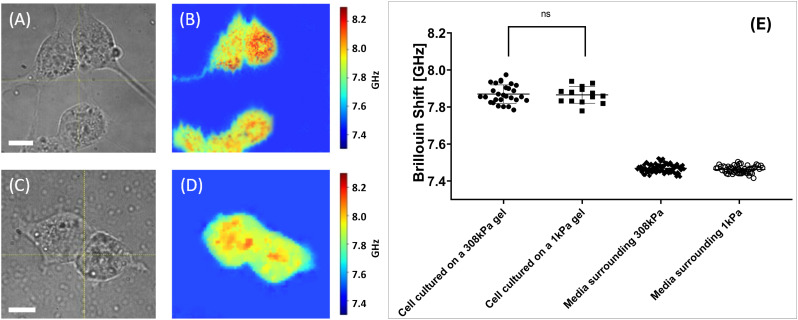
Results of the stiffness measurement experiment with the cells in close proximity. Panels (A) and (C) display wide-field microscope images of an 4T1 fibroblasts on 308 kPa and 1 kPa substrates respectively (60× magnification). White bar 10 μm. Panels (B) and (D) display Brillouin shift value maps of cultures presented in panels (A) and (B) respectively. Panel (E) displays statistics of the Brillouin shift values retrieved from the cells cultured on different substrates. Here, cells grown on 308 kPa hydrogel substrates exhibited the average Brillouin shift value of 7.87 ± 0.05 GHz. Cells grown on 1 kPa hydrogel substrates exhibited the Brillouin shift value of 7.87 ± 0.05 GHz. Data sets are not significantly different (Mann–Whitney test). Brillouin shift values retrieved from the media surrounding the 308 kPa and 1 kPa hydrogels is 7.47 ± 0.02 GHz and 7.46 ± 0.02 GHz. Presented data serves proof that cells cultured on the stiffer hydrogel substrate display a significantly larger Brillouin shift value, corresponding to higher stiffness.

Cells grown in proximity to each other were also imaged, with the results compiled in Fig. 6.

It is well-established that cells communicate with their neighbors through molecular signaling.^54^ Cells secrete signaling molecules into the surrounding environment, which then interact with receptors on the plasma membranes of nearby cells. Through this mechanism, cells are able to detect and respond to the presence of surrounding cells. Previous studies have indicated that the typical distance over which cells can communicate effectively is approximately 250 μm.^55^

Such distances exceeded the field of view of the microscope objective lens used in this study. In our experiment, cells are considered to be standalone if no visible membrane-to-membrane contact is observed within the image field of view. Conversely, cells are classified as being in close proximity if there is visible membrane contact, or if the cells are separated less than 5 μm.

Widefield images presented in Fig. 6A and C reveal that 4T1 murine fibroblasts exhibit distinct morphological characteristics under comparable incubation conditions.

Fibroblasts cultured on 308 kPa hydrogels exhibit elongated protrusions, whereas cells grown on 1 kPa hydrogels assume a more rounded morphology with shorter protrusions. Consistent with the standalone experiments, cells demonstrate improved adhesion to the stiffer substrates.

Cumulative data from the experiment are shown in Fig. 6E. Data was analyzed and presented in the same way as from the experiment with isolated cells described prior. Similarly, the total mean and median Brillouin shift values for these samples were then computed.

Cells grown on 308 kPa substrates exhibited an average Brillouin shift value of 7.87 ± 0.05 GHz with the median value of 7.86 GHz, these values were calculated from 28 cells studied. And cells cultured on 1 kPa substrates displayed an average shift value of 7.87 ± 0.05 GHz and median value of 7.87 GHz, these values were calculated from 14 cells imaged with the Brillouin spectrometer. These findings indicate that cells cultured in close proximity exhibit comparable mechanical stiffness, as evidenced by the similar Brillouin shift values measured across the cell population.

The average Brillouin shift values for the media surrounding the 308 kPa and 1 kPa gels were 7.47 ± 0.02 GHz and 7.46 ± 0.02 GHz respectively, showing the value difference comparable to instrumental error of the system.

The underlying mechanisms responsible for the observed disparity in the correlation between cellular mechanical properties and substrate stiffness, as influenced by cell proximity, are discussed in detail in the following section.

### 3.3. Discussion

The primary objective of this study was to evaluate the capability of Brillouin spectroscopy as a label-free, non-invasive, real-time tool for probing the mechanical response of cells to substrates with varying stiffness. By leveraging Brillouin scattering, which provides quantitative information on cellular viscoelastic properties, we aimed to correlate findings made using other elastographic techniques used for cell elastographic measurements.

Orthogonal methodologies have been employed in several previous studies to investigate the influence of substrate stiffness on cellular mechanical properties, motility, and behavior. These studies have consistently demonstrated that cellular behavior is significantly modulated by variations in substrate stiffness, with cells displaying a pronounced tendency to migrate toward regions exhibiting elevated stiffness. This stiffness-guided migration is thought to be a consequence of cellular mechanosensing mechanisms, wherein mechanoreceptors, such as integrins, engage with the ECM to detect and respond to changes in mechanical cues. These interactions trigger downstream signaling pathways that trigger a dynamic process that involves actin cytoskeleton remodeling. Specifically, cells modulate actin network polymerization, enabling directed movement in response to mechanical gradients.^56^

**Fig. 7:**
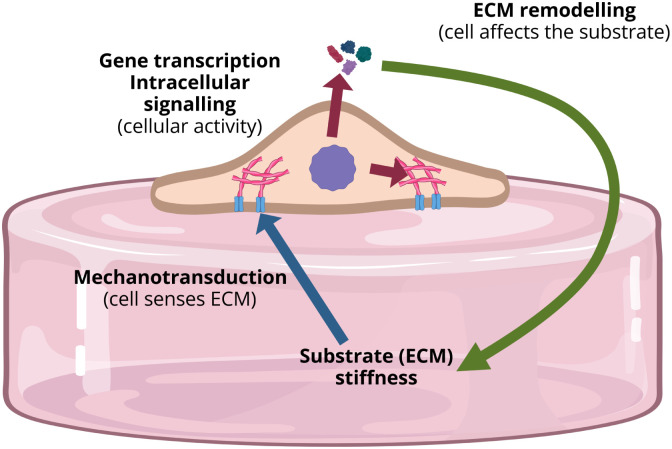
Simplified graphical representation of mechanobiological feedback loop present at cell-ECM interaction. The cell responds to the ECM stiffness *via* integrins in the cell membrane in contact with the substrate (blue arrow). Integrins trigger intracellular activity altering cell behavior, polymerizing actin fibers in the cytoskeleton, stiffening, and excreting ECM-remodeling chemicals (red arrows). Excreted chemicals in turn modify the substrate stiffness (green arrow). Created in https://BioRender.com.

These complex interactions of cell and substrate have been described previously and are illustrated in Fig. 7. The intracellular pathways, are thought to be responsible for dynamic changes in actin fiber polymerization, driving cell motility and overall stiffness.^54,56^

As previously discussed, spontaneous Brillouin scattering is a phenomenon of light scattering on localized longitudinal acoustic waves at the microscopic level.

In the context of cellular mechanics, it was hypothesized that the polymerization of the actin cytoskeleton within the cell body would result in subtle alterations to the microscopic longitudinal acoustic waves inside the cell cytoskeleton. Specifically, the velocity of acoustic wave propagation is expected to be modified due to the increased structural density and crosslinking of the polymerized actin network, which alters the material's overall viscoelastic properties on a microscopic level.

Such changes were anticipated to manifest as a detectable increase in the Brillouin shift value, indicative of enhanced sample rigidity. This increase of Brillouin shift value can be quantitatively captured using our Brillouin spectrometer, allowing to quantify cellular mechanobiological response to ECM stiffness *via* the longitudinal modulus *M*′, defined earlier.

Our experimental results show that cells cultured on the stiff substrate display larger Brillouin shift values, which suggests polymerization of actin fibers in the cytoskeleton. These results are in strong alignment with the anticipated outcomes.

This observation reinforces the relationship between substrate rigidity and cellular mechanical properties, as well as underscores the potential of Brillouin spectroscopy as a non-invasive and label-free tool for the stiffness mapping of live cells.

An additional focus of the study was to investigate changes in cellular mechanobiology in response to cellular proximity. Previous research has indicated that cells placed in sparse configurations exhibit greater traction forces, and consequently increased stiffness, which is attributed to enhanced cellular motility. This phenomenon is driven by dynamic reorganization and polarization of the actin cytoskeleton, which plays a central role in facilitating cell movement. Moreover, prior studies have highlighted the challenges associated with tracking and accurately quantifying the mechanical properties of cells in close proximity to one another, due to the interference of intercellular interactions and overlapping mechanical responses.

On the basis of our observation our observations, cells in close proximity to one another consistently exhibited lower Brillouin shift values under all experimental conditions. Furthermore, no statistically significant differences were observed in the Brillouin shift values between cells cultured on substrates with varying stiffness.

To our knowledge, these findings have not been extensively explored; however, it is hypothesized that perceived softening of the cells may be attributed to the dominance of chemical signaling pathways in close cellular proximity, which may override mechanosensation-driven motility and mechanical responses dependent on substrate stiffness of cells.^57^ This biochemical communication could potentially suppress the dynamic reorganization of the actin cytoskeleton, a process known to be integral to alterations in cellular stiffness.

The other explanation for the observed phenomenon could lie in a more rapid adjustment of the mechanical properties of the substrate to the excretions of the cells (Fig. 7) with a higher cell density.

## 4. Conclusions

Cellular response to the ECM remains a fundamental aspect of modern cellular mechanobiology. Our study underscores the efficacy of Brillouin microspectroscopy as a robust tool for assessing cell culture elasticity. Using this imaging modality we successfully observed a positive correlation between the stiffness of cellular organelles and the stiffness of the ECM. Furthermore, our findings provide evidence that within the context of the cellular environment, close proximity of cells to each other significantly influences their elasticity.

## Conflicts of interest

The authors declare no conflicts of interest.

## Data Availability

The data in this article can be provided upon a reasonable request to the corresponding author.
